# Measuring the impact of screening automation on meta-analyses of diagnostic test accuracy

**DOI:** 10.1186/s13643-019-1162-x

**Published:** 2019-10-28

**Authors:** Christopher R. Norman, Mariska M. G. Leeflang, Raphaël Porcher, Aurélie Névéol

**Affiliations:** 1 LIMSI, CNRS, Université Paris Saclay, Rue du Belvedère, Orsay, 91405 France; 2 Amsterdam Public Health, Amsterdam UMC, University of Amsterdam, Meibergdreef 9, Amsterdam, 1105 AZ the Netherlands; 3 Center for Clinical Epidemiology, Assistance Publique–Hôpitaux de Paris, Hôtel Dieu Hospital; Team METHODS, CRESS, INSERM U1153; University Paris Descartes, 1 place du Parvis Notre-Dame, Paris, 75004 France

**Keywords:** Evidence based medicine, *Machine learning, Natural language processing/*methods, *Systematic review as topic

## Abstract

**Background:**

The large and increasing number of new studies published each year is making literature identification in systematic reviews ever more time-consuming and costly. Technological assistance has been suggested as an alternative to the conventional, manual study identification to mitigate the cost, but previous literature has mainly evaluated methods in terms of recall (search sensitivity) and workload reduction. There is a need to also evaluate whether screening prioritization methods leads to the same results and conclusions as exhaustive manual screening. In this study, we examined the impact of one screening prioritization method based on active learning on sensitivity and specificity estimates in systematic reviews of diagnostic test accuracy.

**Methods:**

We simulated the screening process in 48 Cochrane reviews of diagnostic test accuracy and re-run 400 meta-analyses based on a least 3 studies. We compared screening prioritization (with technological assistance) and screening in randomized order (standard practice without technology assistance). We examined if the screening could have been stopped before identifying all relevant studies while still producing reliable summary estimates. For all meta-analyses, we also examined the relationship between the number of relevant studies and the reliability of the final estimates.

**Results:**

The main meta-analysis in each systematic review could have been performed after screening an average of 30% of the candidate articles (range 0.07 to 100%). No systematic review would have required screening more than 2308 studies, whereas manual screening would have required screening up to 43,363 studies. Despite an average 70% recall, the estimation error would have been 1.3% on average, compared to an average 2% estimation error expected when replicating summary estimate calculations.

**Conclusion:**

Screening prioritization coupled with stopping criteria in diagnostic test accuracy reviews can reliably detect when the screening process has identified a sufficient number of studies to perform the main meta-analysis with an accuracy within pre-specified tolerance limits. However, many of the systematic reviews did not identify a sufficient number of studies that the meta-analyses were accurate within a 2% limit even with exhaustive manual screening, i.e., using current practice.

## Background

The increasing reliance on evidence provided by systematic reviews, coupled with rapidly increasing publishing rates is leading to an increasing need to automate the more labor-intensive parts of the systematic review process [1]. Beyond simply reducing the cost involved in producing systematic reviews, automation technologies, used judiciously, could also help produce more timely systematic reviews.

For systematic reviews of diagnostic test accuracy (DTA), no sensitive and specific methodological search filters are known, and their use is therefore discouraged [2–4]. Consequently, the number of citations to screen in a systematic review of diagnostic test accuracy is often several times higher than for systematic reviews of interventions, and the need for automation may therefore be particularly urgent [5–7].

Methods for automating the screening process have been developed since at least 2006 [8, 9] but have so far seen limited adoption by the systematic review community. While there are examples of past and ongoing systematic reviews using automation, many more use manual screening. Thomas noted in 2013 that in order for widespread adoption to occur, screening automation must not only confer a *relative advantage* (time saved) but must also ensure *compatibility* with the old paradigm, i.e., ensuring that screening automation is equivalent to manual screening [10]. There has been a large number of studies measuring the amount of time saved by automated screening, which may suggest that automation methods are maturing in terms of relative advantage. We are however not aware of any studies focusing on the compatibility aspect: whether automated screening results in the “same” systematic review, and much of the literature to date have implicitly assumed that recall values over 95% are both necessary and sufficient to ensure an unchanged systematic review [8]. In this study, we aim to revisit this hypothesis, which to our knowledge has never been tested.

Among possible automation approaches, only screening prioritization is currently considered safe for use in systematic reviews [8]. In this approach, systematic review authors screen all candidate studies, but in descending order of likelihood of being relevant. It is often assumed that we can achieve some amount of reduction in workload by using screening prioritization [8], but the extent to which this is true has not been evaluated [10]. Screening prioritization can be combined with a cut-off (stopping criterion) to reduce the workload, for example, by stopping screening when the priority scores assigned to remainder of the retrieved studies falls below some threshold. Using cutoffs is generally discouraged since it is not possible to guarantee that no relevant studies remain after the cutoff point and would thus be falsely discarded [8]. However, using cutoffs would likely reduce the workload down to a fraction compared to using screening prioritization alone and may therefore be necessary to fully benefit from screening prioritization.

### Meta-analyses of diagnostic test accuracy

Systematic reviews of diagnostic test accuracy may yield estimates of diagnostic performance with higher accuracy and stronger generalizability than individual studies and are also useful for establishing whether and how the results vary by subgroup [11]. Systematic reviews of diagnostic test accuracy are critical for establishing what tests to recommend in guidelines, as well as for establishing how to interpret test results.

Unlike randomized control trials, which typically report results as a single measure of effect (e.g., as a relative risk ratio), diagnostic test accuracy necessarily involves a trade-off between sensitivity and specificity depending on the threshold for positivity for the test [11, 12]. Diagnostic test accuracy studies therefore usually report results as two or more statistics, e.g., sensitivity and specificity, negative and positive predictive value, or the receiver operating characteristic (ROC) curve. The raw data underlying these statistics is called a 2×2 table, consisting of the true positives, the false positives, the true negatives, and the false negatives for a diagnostic test evaluation.

Meta-analyses of diagnostic test accuracy pool the 2×2 tables reported in multiple DTA studies together to form a summary estimate of the diagnostic test performance. The results of DTA studies are expected to be heterogeneous, and the meta-analysis thus needs to account for both inter- and intra-study variance [12]. This is commonly accomplished using hierarchical random effects models, such as the bivariate method, or the hierarchical summary ROC model [13, 14]. Pooling sensitivity and specificity separately to calculate separate summary values is discouraged, as it may give an erroneous estimate, e.g., a sensitivity/specificity pair not lying on the ROC curve [11].

### Systematic reviews require perfect recall

Systematic reviews are typically expected to identify *all* relevant literature. In the Cochrane Handbook for DTA Reviews [4], we can read:

“Identifying as many relevant studies as possible and documenting the search for studies with sufficient detail so that it can be reproduced is largely what distinguishes a systematic review from a traditional narrative review and should help to minimize bias and assist in achieving more reliable estimates of diagnostic accuracy.”

Thus, the requirement to retrieve all relevant literature may just be a means to achieve unbiased and reliable estimates in the face of, e.g., publication bias, rather than an end in itself. In this context, “as many relevant studies as possible” may be better understood as searching multiple sources, including gray literature, in order to mitigate biases in different databases [4]. Missing a single study in a systematic review could result in the systematic review drawing different conclusions, and recall can therefore, in general, only guarantee an unchanged systematic review if it is 100%. For some systematic reviews, finding all relevant literature may be the purpose of the review, i.e., when the review is conducted to populate literature databases [15]. On the other hand, for systematic reviews addressing diagnostic accuracy or treatment effects, the review may be better helped by identifying an unbiased sample of the literature, sufficiently large to answer the review question [16]. In systematic reviews of interventions, such a sample is often substantially larger than can be identified with the systematic review process [17], but we hypothesize that it can also be substantially smaller.

Of course, many systematic reviews aim not just to produce an accurate estimate of the mean and confidence intervals, but also estimate prevalence, as well as identify and produce estimates for subgroups. Thus, to ensure an unchanged systematic review, we would really need to ensure that the unbiased sample is sufficient to properly answer all aspects of the research question of the review. For instance, an unchanged systematic review of diagnostic test accuracy could require unchanged estimates of summary values, confidence intervals, the identification of all subgroups, and unchanged estimates of prevalence. We will in this study restrict ourselves to measuring the accuracy of the meta-analyses in systematic reviews of diagnostic test accuracy, i.e., the means and confidence intervals of the sensitivity and specificity.

There are multiple potential sources of bias that can affect a systematic review, including publication bias, language bias, citation bias, multiple publication bias, database bias, and inclusion bias [18–20]. While some sources of bias, such as publication bias, mainly occur across databases, others, such as language bias or citation bias may be present within a single database.

However, bias (i.e., only finding studies of a certain kind) is often conflated with the exhaustiveness of the search (i.e., finding all studies). While an exhaustive search implies no bias, a non-exhaustive search may be just as unbiased, provided the sample of the existing literature it identifies is essentially random. If the goal of the systematic review is to estimate the summary diagnostic accuracy of a test, the recall (the sensitivity of the screening procedure) may therefore be less important than the number of studies or total number of participants identified, provided the search process does not systematically find, e.g., English language literature over literature in other languages. However, previous evaluations of automation technologies usually measure only recall or use metrics developed primarily for web searches [6, 7] while side-stepping the (harder to measure) reproducibility, bias, and reliability of the parameter estimation process.

### The impact of rapid reviews on meta-analysis accuracy

Screening prioritization aims to decrease the workload in systematic reviews, while incurring some (presumably acceptable) decrease in accuracy. Similarly to screening prioritization, rapid reviews also seek to reduce the workload in systematic reviews and produce timelier reviews by taking shortcuts during the review process and is sometimes used as an alternative to a full systematic review when a review needs to be completed on a tight schedule [21]. Examples of rapid approaches include limiting the literature search by database, publication date, or language [22].

Unlike screening prioritization, the impact of some rapid review approaches on meta-analyses have been evaluated [22–27]. However, a 2015 review identified 50 unique rapid review approaches, and only a few of these have been rigorously evaluated or used consistently [21]. Limiting inclusion by publication date, excluding smaller trials, or only using the largest found trial have been reported to increase risk of changing meta-analysis results [22]. By contrast, removing non-English language literature, unpublished studies, or grey literature rarely change meta-analysis results [26, 27].

The percentage of included studies in systematic reviews that are indexed in PubMed has been estimated between 84–90%, and restricting the literature search to PubMed has been reported to be relatively safer than other rapid review approaches [22, 24, 28]. However, Nussbaumer-Streit et al. have reported 36% changed conclusions for randomly sampled reviews, and 11% changed conclusions for review with at least ten included studies [23]. The most common change was a decrease in confidence. Marshall et al. also evaluated a PubMed-only search for meta-analyses of interventions and demonstrated changes in result estimates of 5% or more in 19% of meta-analyses, but the observed changes were equally likely to favor controls as interventions [22]. Thus, a PubMed-only search appears to be associated with lower confidence, but not with consistent bias. Halladay et al. have reported significant differences between PubMed-indexed studies and non-PubMed indexed studies in 1 out of 50 meta-analyses including at least 10 studies [24]. While pooled estimates from different database searches may not be biased to favor either interventions or controls, Sampson et al. have reported that studies indexed in Embase but not in PubMed not only exhibit consistently smaller effect sizes, but also reasoned that the prevalence of such studies is low enough that this source of bias is unlikely to be observable in meta-analyses [25].

### Related methods for screening prioritization

The earliest known screening prioritization methods were published in 2006, and a number of methods have been developed since then [9]. Similar work on screening the literature for database curation has been published since 2005 [29, 30]. A wide range of methods (generally from machine learning) have been used to prioritize references for screening, including Support Vector Machines, Naive Bayes, Voting Perceptrons, LAMBDA-Mart, Decision Trees, EvolutionalSVM, WAODE, kNN, Rocchia, hypernym relations, ontologies, Generalized Linear Models, Convolutional Neural Networks, Gradient Boosting Machines, Random Indexing, and Random Forests [6–8, 31]. Several screening prioritization systems are publicly available, including EPPI-Reviewer, Abstrackr, SWIFT-Review, Rayyan, Colandr, and RobotAnalyst [31–35].

The most straightforward screening prioritization approach trains a machine learning model on the included and excluded references from previous iterations of the systematic review, and then uses this model to reduce the workload in future review updates [8]. For natural reasons, this approach can only be used in review updates, and not in new systematic reviews. By contrast, in the *active learning* approach, the model is continuously retrained as more and more references are screened. In a new systematic review, active learning starts with no training data, and the process is typically bootstrapped (“seeded”) by sampling the references randomly, by using unsupervised models such as clustering or topic modeling or by using information retrieval methods with the database query or review protocol as the query [36].

Comparing the relative performance of different methods is difficult since most are evaluated on different datasets, under different settings, and often report different measures. There have been attempts to compare previous methods by replicating reported methods on the same datasets, but the replication of published methods is often difficult or impossible due to insufficient reporting [37]. Another way to compare the relative performance of methods is through the use of a *shared task*, a community challenge where participating systems are trained on the same training data and evaluated blindly using pre-decided metrics [38, 39]. The shared task model removes many of the problems of replication studies and also safeguards against cheating, mistakes, and the cherry-picking of metrics or data, as well as publication bias. The only shared task for screening prioritization we are aware of is the CLEF Shared Task on Technology-Assisted Reviews in Empirical Medicine, focusing on diagnostic test accuracy reviews [6, 7].

The purpose of this study is not to compare the relative performance of different methods, and we will focus on a single method (Waterloo CAL) that ranked highest on most metrics in the CLEF shared task both 2017 and 2018 [6, 7]. As far as we can determine, Waterloo CAL represents the state of the art for new systematic reviews of diagnostic test accuracy (i.e., performed de novo). The training done in Waterloo CAL is also similar to methods currently used prospectively in recent systematic reviews and mainly differs in terms of preprocessing [35, 40, 41].

### Objectives

Our objectives in this study are twofold:

- We aim to retrospectively and prospectively measure the impact of screening automation on meta-analysis accuracy. We will use one single method for analysis in this study, but the criteria should be usable with any screening automation method. We will pay special attention to prospective criteria, since these can also be calculated while the screening is ongoing, and we will examine cut-offs for the prospective criteria that could be used in a prospective setting to bound the loss in accuracy within prespecified tolerance limits.
- We aim to evaluate the (retrospective) 95% recall criterion, which has long been the target to strive for in screening automation, and will test whether this criterion is necessary and sufficient to guarantee an unchanged systematic review. In the case the criterion is not necessary or sufficient, we aim to develop criteria that could be used instead.

## Methods

### Data used in the study

**The Limsi-Cochrane dataset**. This dataset consists of 1939 meta-analyses from 63 systematic reviews of diagnostic test accuracy from the Cochrane Library (the full dataset is available online: DOI: 10.5281/zenodo.1303259) [42]. The dataset comprises all studies that were included in the systematic reviews, from any database or from gray literature, as well as the 2×2 tables (the number of true positives, false positives, true negatives, and false negatives) extracted from each included study by the systematic review authors, grouped by meta-analysis. This dataset can be used to replicate the meta-analyses in these systematic reviews, in full or over subsets of the data, for instance, to evaluate heterogeneity or bias of subgroups.

**The CLEF dataset**. This dataset consists of all references from PubMed considered for inclusion—both those included in the systematic review and those ultimately judged not relevant to the systematic review—in 80 systematic reviews of diagnostic test accuracy also from the Cochrane Library (the full dataset is available online: https://github.com/CLEF-TAR/tar) [6, 7]. Due to the way the data was collected, this dataset only contains references from PubMed, but not from other databases or gray literature. The dataset only includes the PubMed identifiers for each reference and whether the studies were included in the reviews.

**Combined dataset**. For our experiments, we combined the two datasets by collecting the reviews, meta-analyses, and references common to both. In total, this intersection comprises 48 systematic reviews and 1354 meta-analyses of diagnostic tests. All analyses in this study will be based on this intersection unless otherwise specified.

Since the CLEF dataset only includes references from PubMed, the meta-analyses performed in this study will only be based on studies from PubMed. Some meta-analyses may therefore be smaller than they were in the original reviews. The exclusion of studies from other sources than PubMed has been demonstrated to have moderate impact, and no bias on meta-analyses of interventions, and we will make the explicit assumption that the same is true for systematic reviews of diagnostic test accuracy (we are not aware of studies measuring this directly) [22, 24].

Cochrane guidelines for systematic reviews of diagnostic test accuracy discourage drawing conclusions from small meta-analyses but do not offer a specific minimum number of studies required for a meta-analysis [12]. In this study, we will only consider meta-analyses based on three or more studies, because the R package we use (mada) issues a warning when users attempt to calculate summary estimates based on fewer studies [43]. This minimum likely errs on the side of leniency.

We considered as meta-analysis any summary estimate reported individually in the “summary of findings” section of the systematic reviews, regardless of how the estimates were calculated. Thus, we considered meta-analyses of subgroups to constitute distinct meta-analyses, in addition to any meta-analyses of the entire groups of participants. We further considered meta-analyses distinct for the same diagnostic test evaluated with, e.g., multiple cutoff values, whenever these are reported separately in the systematic reviews.

### Automated screening method

We used a previously developed active learning approach to rank all candidate references for each systematic review in descending order of likelihood of being relevant [44]. The method was selected since it was the best performing method for new systematic reviews (performed de novo) in the 2017–2018 Shared Task on Technology Assisted Reviews of Empirical Medicine [6, 7].

We used this ranking to simulate the literature screening process in each systematic review, and for those meta-analyses where at least 3 diagnostic studies were included, we simulated the meta-analysis continuously throughout the screening process. As a control, we performed the same simulation with references screened in randomized order. We assumed that screeners will only stop if prompted to do so by the system. If not prompted to stop, the screeners will continue screening until all candidate studies have been screened.

We use a variant of active learning that has demonstrated good performance in systematic reviews of diagnostic test accuracy as well as in article discovery in the legal domain [45]. In this method, we start with an artificial training set, where we use the protocol of the review as an single initial positive training example (seed document). This artificial seed document is discarded as soon as real positive examples are found. We select 100 references randomly from the evaluation set and use these as negative examples, regardless of whether they are really positive or negative. In each iteration, new “negative” examples are randomly selected in this way such that the total number of negative examples is always at least 100. Following Cormack and Grossman, we show *B* references to the screener in each iteration, where *B* is initially set to 1, then increased by ⌈*B*/10⌉ in each subsequent iteration [46].

To train, we use logistic regression with stochastic gradient descent on bigrams and unigrams extracted from the text in titles and abstracts.

### Evolution of a summary estimate

We define the *effort* in a screening process as the number of candidate studies screened so far. Thus, we will for simplicity assume that screening a single article will always incur the same cost.

To measure the reliability of a summary estimate, we define the *loss* at each timestep as the absolute distance to its “true” value, similarly to previous work on the evolution of heterogeneity estimates by Thorlund et al. [47]. To obtain a scalar loss score for a sensitivity/specificity pair, we use the euclidean *L*_2_ distance to the true value. That is, given a true sensitivity/specificity (*μ*,*ν*), then for any estimate $(\hat {\mu }, \hat {\nu }),$ we define its *L*_2_ loss as

$$L_{2}(\hat{\mu}, \hat{\nu}) = \sqrt[]{(\mu - \hat{\mu})^{2} + (\nu - \hat{\nu})^{2}}$$

Similarly to Thorlund et al., we used the final estimate over all relevant studies as a good approximation of the “truth” [47]. This however assumes that the number of relevant studies is sufficiently large that the final summary estimates have converged and are stable.

**Fig. 1:**
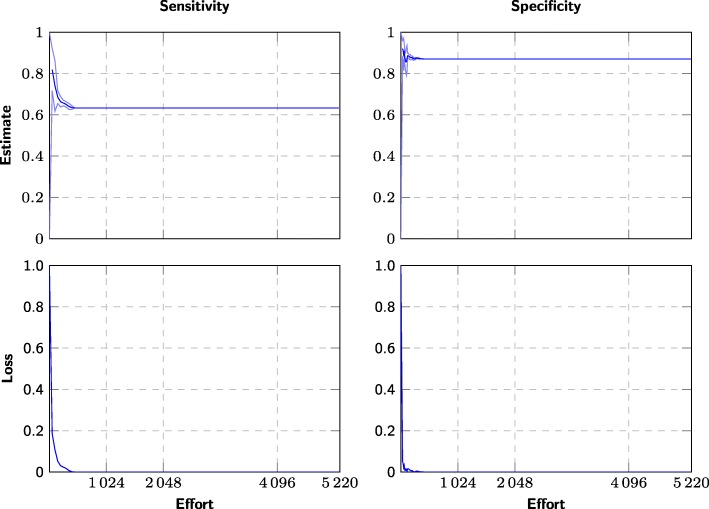
Example of effort/loss curve for a single meta-analysis using screening prioritization. The evolution of the sensitivity and specificity estimates for one diagnostic test “CD008803 1 GDx: inferior average” (*n*=48), where the candidate studies are screened using screening prioritization. The *x*-axis measures the number of screened studies (effort) and the the *y*-axis measures the summary estimates at the 25%, 50%, and 75% percentiles over 20 simulated screenings using screening prioritization. We also plot the difference to the “true” values (bottom)

**Fig. 2:**
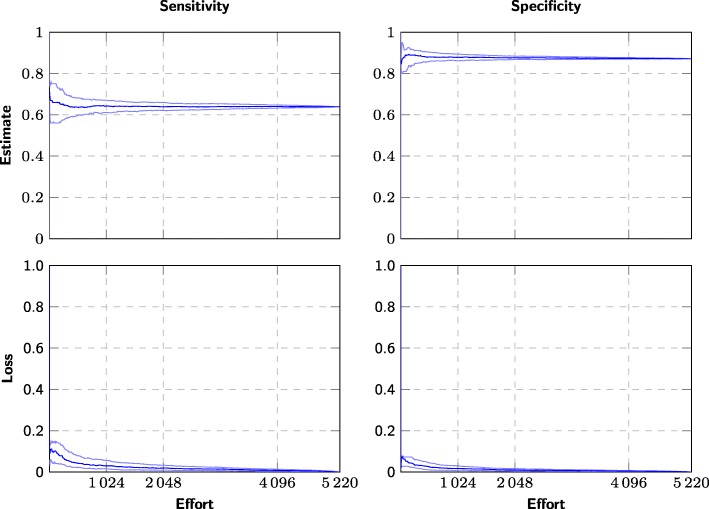
Example of effort/loss curve for a single meta-analysis using randomized order. The evolution of the sensitivity and specificity estimates for one diagnostic test “CD008803 1 GDx: inferior average” (*n*=48), where the candidate studies are screened in arbitrary order. The *x*-axis measures the number of screened studies (effort) and the the *y*-axis measures the summary estimates at the 25%, 50%, and 75% percentiles over 400 simulated screenings using arbitrary (pseudorandom) order. We also plot the difference to the “true” values (bottom)

Conventionally, the screening process first identifies all relevant studies, and the summary estimates are only estimated after the screening process has finished. However, nothing prevents systematic review authors from calculating an estimate as soon as some minimum number of studies have been identified, and then recalculate this estimate every time a relevant article is discovered (see Figs. 1–2). Continuously updated, we should expect the estimate to be unreliable at first, but converge to its true value, and equivalently, the loss to approach zero.

### Finding a balance between loss and effort

To search for an optimal balance between loss and effort, we consider two types of stopping criteria, retrospective and prospective.

*Retrospective stopping criteria* (cutoffs) are evaluated on the effort/loss curve (Figs. 1–2) or using other information only available after screening has finished, and these criteria can therefore only be applied retrospectively. While we cannot use these criteria to decide when to stop the screening, we can use them for evaluation, i.e., to retrospectively see where we could theoretically have interrupted screening without impacting the accuracy of the summary estimate.

*Prospective stopping criteria* can be evaluated without knowing the final estimates or the total number of relevant studies among the candidates and can therefore be used for decision support in a live systematic review.

#### Retrospective stopping criteria

##### Recall (R)

The recall, or the sensitivity of the screening procedure, measures what fraction of the relevant studies were identified by the screening procedure. Commonly, only very high values are considered acceptable (*R*=95*%* and *R*=100*%*), but values as low as *R*=55*%* have been considered [48].

This is one of the only measures commonly used in previous literature [8] and forms the basis for evaluation measures such as WSS@95 [9]. Common performance metrics such as WSS@95 evaluates the theoretical workload reduction if screening were somehow to be interrupted after identifying 95% of all relevant studies. However, it is not possible to know when this point has been reached during a systematic review, since it is not possible to know the number of relevant studies before screening all references.

##### Knee/elbow method

**Fig. 3:**
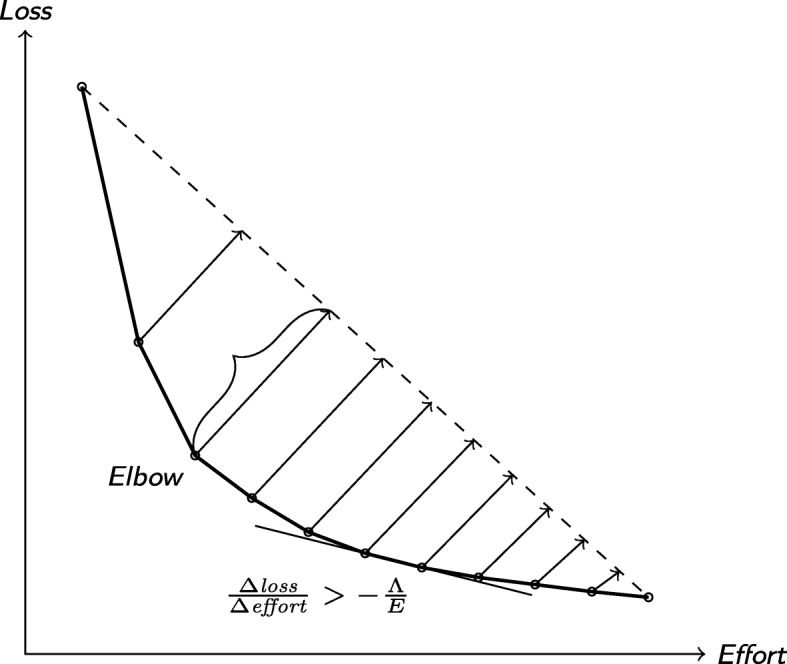
The elbow algorithm and the slope criterion

We here stop at the “elbow” point on the effort/loss curve (Fig. 3). This is a point on the curve corresponding to the optimal point in terms of balance between effort and estimated precision.

Multiple definitions of the elbow point exist. We here use the definition due to Satopää et al. [49], which is easy to implement and robust against noise. Under this definition, the knee point on the effort/gain curve is the one furthest from a straight line drawn from the first and last points on the curve.

##### Loss/effort

We here stop at the point on the effort/loss curve where we would have needed to screen at least *E* references to further reduce the *L*_2_ loss by at least *Λ* (Fig. 3).

This corresponds to the first consecutive pair of points (*e*_*t*−1_,*λ*_*t*−1_), (*e*_*t*_,*λ*_*t*_) on the convex hull of the effort/loss curve such that

$$\frac{\Delta\text{loss}_{t}}{\Delta\text{effort}_{t}} = \frac{\Delta\lambda_{t}}{\Delta e_{t}} = \frac{\lambda_{t} - \lambda_{t-1}}{e_{t} - e_{t-1}} > -\frac{\Lambda}{E}$$

Since we can only calculate the loss after the screening has finished, we can only apply the criterion retrospectively in this study.

The same stopping criterion has been used in similar applications, for instance, for determining when all themes have been identified in ecological surveys [50]. However, the effort/loss curve does not move in only one direction, since adding a single study frequently shifts the estimate away from the truth. Whenever this happens, $\frac {\Delta \lambda _{t}}{\Delta e_{t}}$ will change signs and immediately trigger the condition. To prevent this from happening, we take the convex hull of the curve, which makes the curve monotonously decreasing.

#### Prospective stopping criteria

##### Number of relevant studies retrieved

We here stop as soon as we have identified *n* relevant studies.

##### Found/effort

This criterion is conceptually similar to the loss/effort criterion, except that we use the number of relevant studies found instead of the loss. We here stop at the point where we have to screen at least *E* references to find *F* additional relevant studies [50].

This corresponds to the first consecutive pair of points (*e*_*t*−1_,*f*_*t*−1_), (*e*_*t*_,*f*_*t*_) on the the found/effort curve such that

$$\frac{\Delta\text{found}_{t}}{\Delta\text{effort}_{t}} = \frac{\Delta f_{t}}{\Delta e_{t}} = \frac{f_{t} - f_{t-1}}{e_{t} - e_{t-1}} < \frac{F}{E}$$

Unlike the loss/effort, the number of found relevant studies is monotonously increasing and we therefore do not need to take the convex hull of the found/effort curve.

This criterion is equivalent to stopping when we have not encountered a new relevant study among the last *E*/*F* candidate studies screened, and the criterion will therefore always incur a constant effort penalty equal to *E*/*F*.

##### Displacement

Every time we identify an additional relevant study, we calculate how much the sensitivity and specificity estimates change when the study is included in the meta-analysis. That is, if two consecutively identified relevant studies were identified at time steps *t* and *t*−1, and *s*_*t*_=(*μ*_*t*_,*ν*_*t*_) and *s*_*t*−1_=(*μ*_*t*−1_,*ν*_*t*−1_) are the summary estimates of sensitivity and specificity at these time points, then we define the displacement at time *t* as

$$\Delta\lambda_{t} = \sqrt[]{(\mu_{t} - \mu_{t-1})^{2} + (\nu_{t} - \nu_{t-1})^{2}}$$

To make the results less sensitive to noise, we will mainly consider the moving average (MA) of the displacement with window size 2 (abbreviated MA2).

This criterion can only be calculated if a summary estimate can be calculated and is therefore undefined until at least three relevant studies have been found.

##### Displacement (loocv)

For any set of of references, we calculate the *leave-one-out cross-validated* (LOOCV) [51] displacement as the median displacement when excluding each reference from the summary estimate calculations.

That is, consider that a set of studies *S* has been identified at some point in the screening process, where (*μ*,*ν*) is the summary estimate that would result when calculated based on all studies in *S*. Further, let (*μ*_*s*_,*ν*_*s*_) be the summary estimate that would result from excluding a single study *s*∈*S*. Then, we define the LOOCV displacement as

$$\Delta\lambda_{S} = \underset{s \in S}{\text{median}}\left[\sqrt[]{(\mu - \mu_{s})^{2} + (\nu - \nu_{s})^{2}}\right]$$

This criterion can only be calculated if a summary estimate can be calculated and is therefore undefined until at least three relevant studies have been found.

### Calculation of summary statistics

To calculate the summary estimates, we used the reitsma function from the mada R package [43], which implements the Reitsma bivariate random effects model [13].

## Results

### Characteristics of the systematic reviews

In the 63 systematic reviews in the Limsi-Cochrane dataset, the minimum number of meta-analyses was 1 (3 reviews), the mode was 2 (11 reviews), the median was 6, and the maximum was 170.

We used the combined dataset for all analyses. This dataset comprises 48 systematic reviews and 1354 meta-analyses of diagnostic test accuracy, but only 400 of the meta-analyses were based on at least 3 primary studies in PubMed and thus included in our analysis. Ninety-six of the meta-analyses were based on ten or more studies in PubMed. While we only consider studies from PubMed in this study, which decreases the number of studies per meta-analysis, the large majority of meta-analyses in the original systematic reviews were based on only one or two studies collected from multiple databases [42].

The small size of the meta-analyses were reflected in the number of times the stopping criteria triggered. With cutoff set to 1 relevant per 500 screened, the found/effort criterion would have triggered for 277/400 meta-analyses and for all meta-analyses in 30/48 systematic reviews (ranked, found/effort (1/500) in Table 1). With cutoff set to 1 relevant per 2000 screened, it would have triggered for 174/400 meta-analyses and for all meta-analyses in 17/48 systematic reviews (ranked, found/effort (1/2,000) in Table 1). With cutoff set to 0.02, the displacement criterion would have triggered for 91/400 meta-analyses or for all meta-analyses in 4/48 systematic reviews (ranked, displacement MA2 (0.02) in Table 1). With cutoff set to 0.005, it would have triggered for 35/400 meta-analyses and for all meta-analyses in no systematic review (ranked, displacement MA2 (0.005) in Table 1).

**Table 1:** Average measured loss for each criterion, measured for all tests where the criteria triggered

		Triggered		Effort		Recall	*L*^2^ loss	Sensitivity loss			Specificity loss		
Criterion type	Criterion	MA	SR	abs	perc			mean	lb	ub	mean	lb	ub
Ranked													
Retrospective	Recall (95%)	41	1	5494.122	0.489	0.960	0.001	0.001	0.001	0.001	0.000	0.000	0.000
	Knee/elbow	314	30	120.752	0.047	0.299	0.056	0.040	0.174	0.086	0.029	0.221	0.038
	Loss/effort (0.02/1000)	314	30	259.570	0.104	0.527	0.023	0.018	0.140	0.053	0.010	0.170	0.026
	Loss/effort (0.015/1000)	314	30	271.589	0.108	0.539	0.022	0.018	0.136	0.052	0.009	0.168	0.025
	Loss/effort (0.01/1000)	314	30	297.064	0.113	0.546	0.021	0.016	0.136	0.049	0.009	0.166	0.023
Prospective	Found/effort (1/500)	277	30	811.220	0.268	0.770	0.012	0.008	0.011	0.014	0.006	0.020	0.003
	Found/effort (1/1000)	254	22	1338.465	0.386	0.783	0.007	0.005	0.006	0.005	0.003	0.007	0.002
	Found/effort (1/2000)	174	17	2330.448	0.424	0.815	0.002	0.002	0.002	0.002	0.001	0.002	0.000
	Relevant found (*n*= 20)	41	1	162.732	0.050	0.565	0.023	0.020	0.026	0.022	0.007	0.014	0.008
	Relevant found (*n*= 15)	57	3	140.000	0.039	0.534	0.029	0.024	0.029	0.026	0.010	0.020	0.011
	Relevant found (*n*= 10)	96	4	109.042	0.044	0.529	0.031	0.024	0.039	0.028	0.013	0.027	0.014
	Displacement MA2 (0.005)	35	0	344.714	0.086	0.667	0.014	0.012	0.015	0.012	0.005	0.011	0.005
	Displacement MA2 (0.010)	57	2	280.000	0.065	0.578	0.018	0.016	0.021	0.018	0.005	0.013	0.008
	Displacement MA2 (0.015)	74	3	205.189	0.059	0.538	0.024	0.019	0.030	0.021	0.008	0.018	0.012
	Displacement MA2 (0.020)	91	4	124.099	0.044	0.511	0.029	0.024	0.034	0.025	0.010	0.022	0.013
	Displacement LOOCV (0.005)	42	0	131.667	0.050	0.538	0.021	0.019	0.030	0.016	0.006	0.017	0.008
	Displacement LOOCV (0.010)	90	3	192.356	0.054	0.461	0.030	0.026	0.038	0.025	0.010	0.021	0.013
	Displacement LOOCV (0.015)	130	5	161.923	0.063	0.427	0.035	0.028	0.042	0.030	0.013	0.029	0.015
	Displacement LOOCV (0.020)	152	5	172.947	0.053	0.370	0.042	0.034	0.054	0.034	0.016	0.033	0.019
Randomized													
Retrospective	Recall (95%)	36	1	6956.583	0.961	0.959	0.002	0.002	0.002	0.002	0.000	0.001	0.000
	Knee/elbow	314	30	1658.322	0.299	0.203	0.064	0.042	0.208	0.103	0.039	0.228	0.051
	Loss/effort (0.02/1000)	309	27	1315.816	0.351	0.264	0.047	0.036	0.269	0.101	0.022	0.333	0.049
	Loss/effort (0.015/1000)	309	27	1429.359	0.364	0.285	0.043	0.033	0.249	0.097	0.020	0.309	0.046
	Loss/effort (0.01/1000)	309	27	1552.871	0.390	0.313	0.040	0.030	0.237	0.093	0.018	0.299	0.043
Prospective	Found/effort (1/500)	218	16	1854.106	0.374	0.169	0.112	0.073	0.329	0.143	0.063	0.418	0.061
	Found/effort (1/1000)	151	10	2942.583	0.465	0.237	0.097	0.062	0.268	0.113	0.055	0.338	0.045
	Found/effort (1/2000)	68	6	5097.559	0.493	0.272	0.082	0.052	0.239	0.083	0.042	0.294	0.022
	Relevant found (*n*= 20)	41	1	5106.000	0.574	0.565	0.022	0.018	0.023	0.021	0.008	0.013	0.010
	Relevant found (*n*= 15)	57	3	4288.070	0.550	0.534	0.024	0.020	0.026	0.025	0.010	0.015	0.013
	Relevant found (*n*= 10)	93	4	3275.194	0.537	0.517	0.032	0.024	0.039	0.028	0.015	0.025	0.016
	Displacement MA2 (0.005)	33	0	6478.970	0.663	0.644	0.016	0.014	0.017	0.014	0.006	0.011	0.009
	Displacement MA2 (0.010)	54	2	4395.889	0.537	0.519	0.023	0.019	0.027	0.021	0.009	0.016	0.012
	Displacement MA2 (0.015)	74	3	4103.730	0.533	0.505	0.025	0.021	0.030	0.023	0.010	0.017	0.011
	Displacement MA2 (0.020)	87	4	3478.276	0.509	0.485	0.026	0.022	0.034	0.024	0.010	0.020	0.013
	Displacement LOOCV (0.005)	40	1	5525.525	0.557	0.537	0.017	0.014	0.020	0.014	0.008	0.014	0.007
	Displacement LOOCV (0.010)	87	3	3928.483	0.498	0.454	0.027	0.022	0.037	0.022	0.009	0.022	0.011
	Displacement LOOCV (0.015)	130	4	2902.831	0.475	0.415	0.038	0.031	0.048	0.030	0.014	0.032	0.015
	Displacement LOOCV (0.020)	154	5	2808.455	0.453	0.396	0.041	0.035	0.051	0.034	0.014	0.035	0.016

Triggered signifies the number of meta-analyses (MA, maximum 400) for which the criterion triggered and the number of systematic reviews (SR, maximum 48) where the criterion triggered for all meta-analyses. Effort signifies the absolute and relative number of references needed to be screened before triggering the stopping criteria. Recall signifies the percentage of relevant studies identified when the stopping criterion triggered. The loss in sensitivity and specificity are measured as the difference to the final estimates at the criterion threshold. We also include the the difference between the measured lower and upper bounds of the 95% confidence intervals and their final estimated values (lb, ub)

### How many studies does it take to make a meta-analysis?

**Fig. 4:**
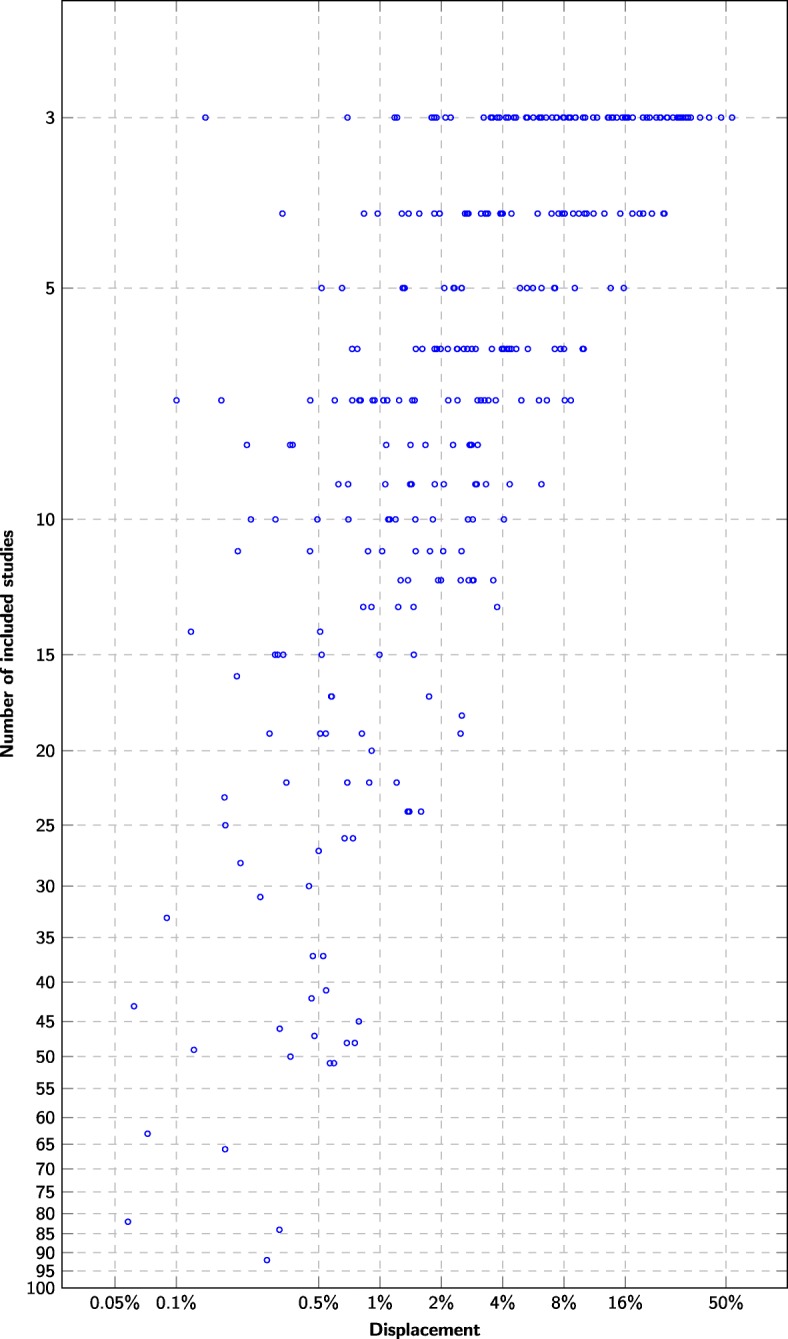
Displacement versus number of relevant primary studies The *x*-axis denotes how much the estimate changed when the last relevant primary study was included (*L*_2_ distance between successive sensitivity/specificity pairs). The *y*-axis denotes the total number of relevant primary studies found for the diagnostic test

The displacement when including the last relevant study in the meta-analyses decreases with the total number *n* of studies included in the meta-analysis (Fig. 4). The last primary study added to the summary estimate calculations displace the estimates by 16 percentage points or less for *n*≥5, by 4 points or less for *n*≥10, by 2 points or less for *n*≥20, and by 1 points or less for *n*≥50.

There is a moderately strong correlation (Pearson *r*=0.54) between the last displacement and the *L*_2_ loss at each summary estimate update. The correlation can be made somewhat stronger by taking the moving average over the last few successive summary estimate updates to cancel out some of the spurious values (MA2: *r*=0.59, MA3: *r*=0.60, MA4: *r*=0.59, MA5: *r*=0.58). Averaging the displacement using leave-one-out cross-validation [51] gives similar correlation to MA3 (*r*=0.60).

### Contribution of screening prioritization

Screening prioritization requires screening a much smaller number of candidate references to reach the cutoff point for all criteria, particularly for prospective criteria. For instance, identification of at least ten relevant primary studies for each applicable meta-analysis would be reached after screening an average of 4.4% of the candidate studies, while we would have needed to screen an average of 53.7% of the candidate studies in randomized order to achieve the same (relevant found (*n*=10) in Table 1). To identify 20 relevant studies for each meta-analysis, it would have been necessary to screen an average of 57.4% of the references in random order, but only 5.0% using screening prioritization (relevant found (*n*=20) in Table 1).

For all criteria except the found/effort, the estimation error is similar at the cutoff point for prioritized screening and screening in random order.

On average, the displacement threshold criterion and the number of relevant found exhibit roughly similar behavior in terms of accuracy and efficiency. In Table 1, we see that if we stop after finding ten relevant studies (criterion: “relevant found (*n*=10)”), we would misestimate the mean sensitivity by approximately 2.4 percentage point and the mean specificity by approximately 1.3 percentage point. If we stop after observing a mean 0.02 displacement over the last two updates (criterion: “displacement MA2 (0.02)”), we would also have needed to screen 4.4% of the candidate studies on average, and we would have misestimated the mean sensitivity by approximately 2.4 percentage point and the mean specificity by 1.0 percentage point.

Stricter thresholds allow trading a higher screening workload for lower estimation error. For instance, stopping after finding 20 relevant studies (criterion: “relevant found (*n*=20)”) leads to screening 5.0% of the candidate studies on average and misestimates the mean sensitivity by approximately 2.0 percentage point and the mean specificity by approximately 0.7 percentage point. Similarly, stopping after observing a mean 0.005 displacement over the last two updates (criterion: “displacement MA2 (0.005)”) leads to screening 8.6% of the candidate studies on average and misestimates the mean sensitivity by approximately 1.2 percentage point and the mean specificity by approximately 0.7 percentage point.

**Fig. 5:**
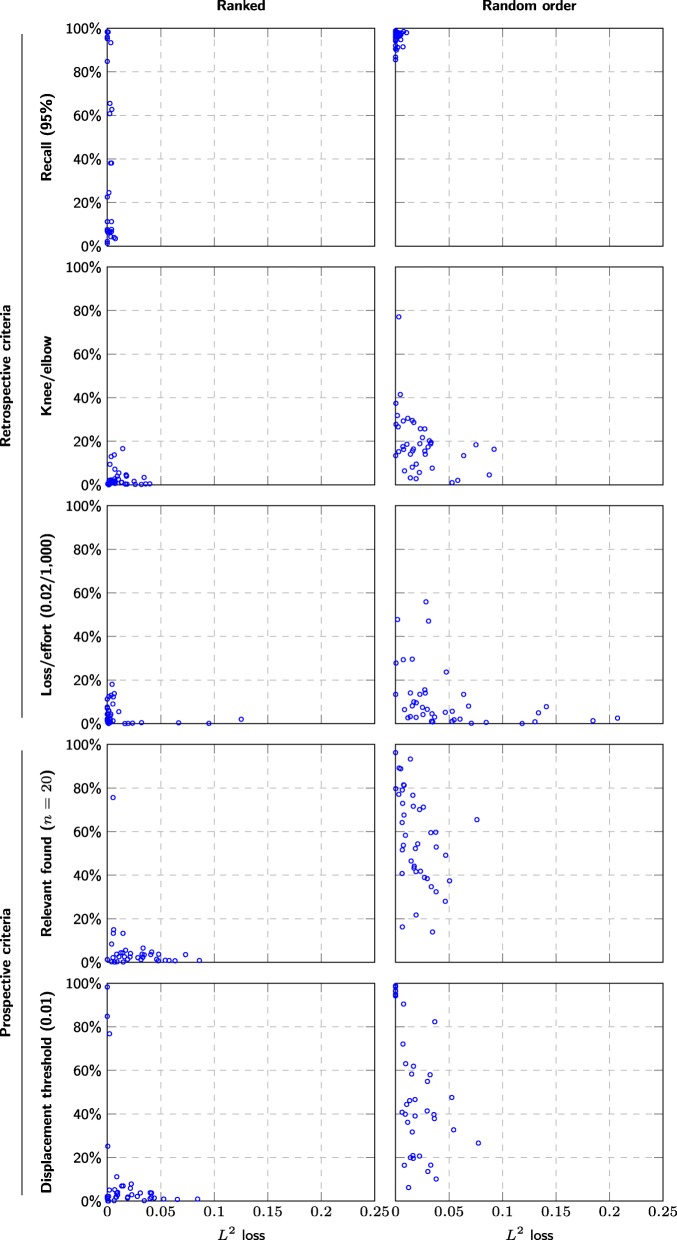
Comparison between stopping criteria effort (*y*-axis) versus *L*^2^ distance to final summary estimate (*x*-axis) for each stopping criteria in the meta-analyses. We only included meta-analyses based on at least 20 studies, so that the criteria were applicable to all meta-analyses and consequently that all data points occur in all scatterplots. This is limited by the relevant found criterion, which only makes sense for meta-analyses based on at least 20 studies

However, while the average discrepancy is only 2 percentage point, the results vary greatly between meta-analyses, and the discrepancy for a given meta-analysis may be as high as 8 percentage point, even with a conservative threshold (Fig. 5).

## Discussion

By monitoring the moving average of the displacement, we were able to estimate the current precision of the diagnostic test accuracy estimates through the screening process. However, the meta-analyses of diagnostic test accuracy were accurate within 2% only for meta-analyses including at least 20 studies (Fig. 4). A criterion to interrupt screening once the displacement falls below 2% would consequently have triggered in 91/400 meta-analyses (Table 1). Many meta-analyses had poor accuracy even when based on all relevant studies (Fig. 4).

### Estimates converge faster using screening prioritization

Screening prioritization identifies most or all relevant primary studies much earlier in the screening process compared to randomized order (Fig. 5). The rate of identification of relevant studies will generally be high initially, before dropping down to a trickle. This rate can be used either to estimate how many relevant studies exist among the candidates [45] or directly as a stopping criteria (cf. found/effort in Table 1). When screening in randomized order, the gaps between successive relevant studies is likely to be large, with highly variable size, which makes it more difficult to estimate the identification rate or the total number of relevant studies. Consequently, the found/effort criterion interrupts too prematurely in randomized order leading to higher loss for sensitivity, specificity, and their associated confidence intervals, for all evaluated cutoffs (Table 1, bottom section).

We can also observe that the summary estimates converge to their final values much more quickly and reliably than when screening in arbitrary order (Figs. 1 and 2). In other words, screening prioritization allows producing almost the same estimates with reduced effort—the problem is knowing whether it is safe to interrupt the screening prematurely. However, screening prioritization may allow meta-analyses to be started after screening a few percent of the candidate references. Even if the authors of the systematic review decide that all references need to be screened to ensure that nothing is missed, the meta-analysis may be conducted in parallel with screening the remaining references and can later be updated to account for any additional studies found.

### Sufficiently large meta-analyses can be stopped prematurely

For any individual summary estimate, we can have two outcomes:

1. The systematic review fails to identify sufficient evidence, and the estimates produced by the published systematic review may in fact be biased or unreliable due to the insufficient amount of evidence.
2. The estimate is unbiased and reliable at some point in the screening process. Continuing the screening process is unlikely to change the precision of the estimate (cf. Fig. 1), and the effort could arguably be spent elsewhere.

The systematic review process implicitly assumes the borderline case between these two, where the estimate becomes unbiased and reliable only and exactly at the end of the screening. Our results suggest this may not be an unreasonable assumption when screening in random order—the displacement fell below a tolerance of 0.1 only during the last 10% of the screened references for 10 out of 41 meta-analyses based on at least 10 studies (random, displacement threshold (0.01) in Fig. 5). However, the same was only true for 1 out of 41 meta-analyses when using ranked order (ranked, displacement threshold (0.01) in Fig. 5).

In case 1, we could arguably stop screening (and possibly refine the database search) as soon as it becomes clear that a sufficient number of relevant studies cannot be retrieved. We cannot know with absolute certainty how many remaining studies exist for us to find. However, the found/effort curve will typically be convex when the candidate list is ranked, and extrapolating from its current slope therefore provides a probabilistic upper bound of the number of remaining studies [45].

Case 2 assumes a sufficiently large amount of evidence to base the summary estimates upon. Then, as additional evidence is accumulated, the summary estimate will converge to its true value. The value of additional evidence will drop accordingly as the estimate becomes increasingly stable.

We previously estimated the average discrepancy when replicating summary estimates in the systematic reviews at approximately 2 percentage point [42], and we can take this as a minimum requirement for estimation accuracy. On average, we can achieve the same or better estimation accuracy with the displacement criterion with a cutoff of 0.01 or lower or with the found/effort criterion with a cutoff of 1/500 or lower.

### Data saturation is seldom reached in DTA systematic reviews

We observe a consistent positive relationship between meta-analysis size and the accuracy of the estimates (Fig. 4). The least accurate diagnostic test accuracy estimates occurred for meta-analyses of three included studies and were accurate only within roughly 50% of their final values (Fig. 4). The vast majority of estimates were not accurate within 2% at the end of the screening process. These results mirror the work of Wetterslev et al, who have previously observed that most Cochrane systematic reviews of interventions are insufficiently powered to even detect or reject large intervention effects [17]

Our stopping criteria based on displacement will only interrupt the screening process once the estimates have stabilized due to data saturation. If data saturation fails to occur because too few studies exist to find, the screening will not be interrupted. We can however also interrupt screening if it becomes clear that no further studies will be uncovered by the screening process, i.e., by using a stopping criterion like found vs effort.

**Fig. 6:**
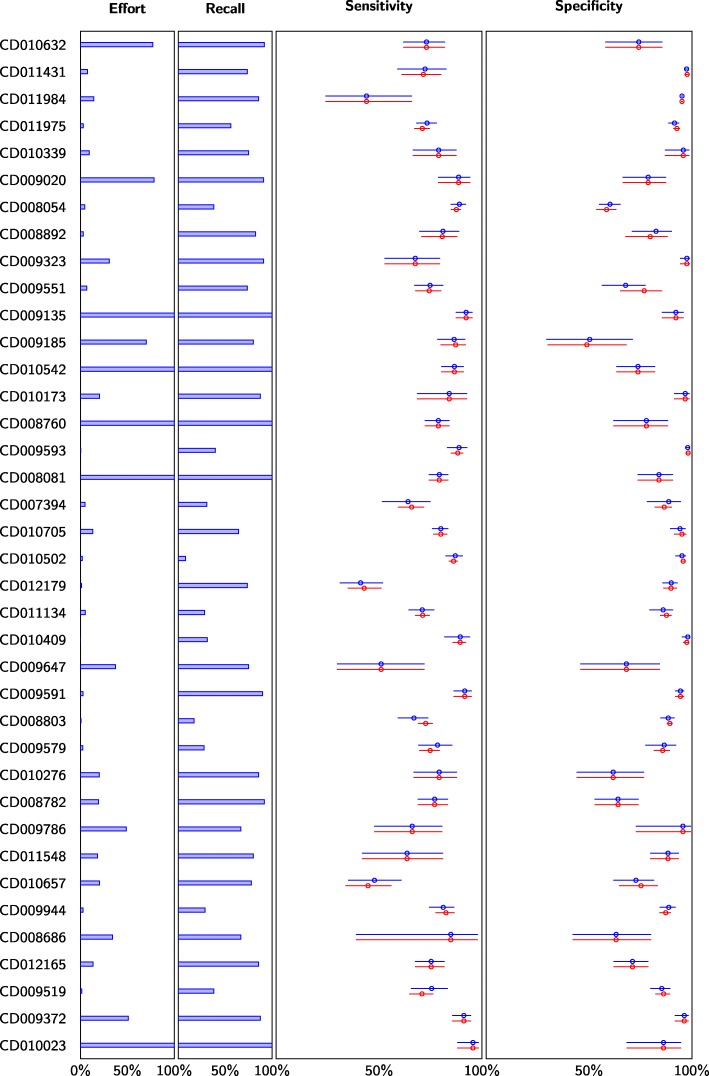
The impact of screening prioritization and stopping criteria on meta-analyses. Difference in meta-analysis results for the largest meta-analysis in each systematic review using a combination of stopping criteria (displacement (0.01) OR relevant (*n* = 15) OR found/effort (1/1000)). Ten systematic reviews did not include any meta-analysis based on three or more studies (in PubMed) and were therefore excluded from the results. Effort denotes the fraction of candidate references screened. Recall denotes the fraction of identified relevant studies. Blue data points correspond to the simulated results using early stopping. Red data points correspond to results without early stopping, i.e., equivalent to current practice (which would have 100% effort and 100% recall)

For instance, using a combination of stopping criteria (displacement (0.01) OR relevant (*n* = 15) OR found/effort (1/1000)) would have reduced the screening effort by 21.5–99.9285% (mean 81.7%, median 90.56%) for the main meta-analysis in 33 out of 38 systematic reviews with an average 1.2% estimation error (Fig. 6). The five systematic reviews where the effort would not have been reduced were among the smallest with a total number of candidate studies ranging from 64 to 981. Ten systematic reviews performed no meta-analysis with at least three studies in PubMed and were therefore excluded from this analysis.

### External validity

We have presented seven criteria and have evaluated how these perform when using logistic regression for ranking and when using random order. We expect these criteria to generalize differently if used with other methods.

The *L*_2_ loss guarantees presented for the recall, the relevant found, and the displacement (either with MA2 or LOOCV) only depend on the relative order of the relevant studies and is otherwise independent of where in the ranking the relevant studies occur. In other words, whether our results for these criteria extend to other methods only depends on how the method orders the relevant studies. In this study, we demonstrate that using these criteria with logistic regression results in the same *L*_2_ loss compared to random order, and thus that logistic regression does not bias the meta-analyses compared to random order. In light of this, we expect these criteria to yield similar *L*_2_ loss for any ranking method that is similarly unbiased.

The knee/elbow criterion, the loss/effort criterion, and the found/effort criterion all depend on the relative order of all studies, both relevant and non-relevant, and can therefore be expected to give different results depending on the strength of the ranking method. We can observe this in Fig. 5, where the knee/elbow criterion and the loss/effort criterion result in larger and more frequent *L*_2_ losses for random order than for ranked order. The found/effort criterion breaks down entirely for random order and yields unacceptably large *L*_2_ losses (see randomized: found/effort in Table 6). In light of this, the parameters we use for these criteria thus cannot be assumed to yield the same *L*_2_ losses for other ranking methods and would need to be recalibrated when used with other methods.

In this study, we have only considered meta-analyses with at least three included studies. However, the prospective criteria are conservative and will simply not trigger when used in a systematic review where there are only two or less studies to find. The only exception is the found/effort criterion, but this criterion can easily be modified so that it is ignored before at least three relevant studies have been found.

### Recommendations

We explicitly refrain from recommending specific stopping criteria or specific cutoff values, since there is no one size that fits all systematic reviews—the criteria and their parameters need to be decided to suit the purposes of the review. If automation is adopted in a systematic review, acceptable tolerances should be decided as part of the protocol, and the protocol should include a strategy to ensure that the tolerance criteria will be satisfied.

We recommend that several stopping criteria be monitored in parallel and that screening is interrupted only once criteria for all necessary aspects of the systematic review are satisfied. In this study, we focus on the accuracy of the main meta-analysis—similar criteria should also be specified for all other aspects deemed necessary for the review, such as the identification of all subgroups or estimates of prevalence of the diagnosed condition.

Specifically, to monitor the accuracy of the sensitivity and specificity estimates, we recommend the use of:

- The displacement MA2 criterion, set to half the required tolerance
- The displacement LOOCV criterion, set to half the required tolerance
- The relevant found criterion, set convervatively (15 at a minimum)

The MA2 and LOOCV displacement yield similar information and do not need to be monitored simultaneously. The LOOCV variant underestimated the loss in our experiments more than the MA2 and triggered more often with larger average *L*_2_ loss, and we therefore recommend the MA2 variant over LOOCV. On average, both variants overestimated the final *L*_2_ loss and we recommend the displacement be interpreted with this in mind.

These criteria triggering mean that the current estimate is accurate within a given tolerance and that further studies are unlikely to change the estimates, even if a large number of relevant studies still exist to find. These criteria can also be used with randomized screening and likely also for any screening prioritization method that does not bias the order of the relevant studies. If the displacement criterion is infeasible to calculate, the relevant found criterion can be used alone, but it may be difficult to infer meta-analysis accuracy from the number of relevant studies included.

- The loss/effort criterion with a conservative parameter setting (1/1000 or stricter)

This criterion triggering is an indication that no further studies exist to find. This criterion should be treated with more caution than the other criteria. In particular, the criterion depends on the strength of the screening prioritization method and can trigger prematurely, e.g., if the method struggles to find some subset of the relevant studies or if the screening prioritization method is generally poor.

The found/effort criterion is also more likely to trigger prematurely if the total number of relevant studies is low. Therefore, we also recommend not using this criterion until some minimum number of studies have been identified (three appear to be a safe choice for the current setting and the current method).

### Limitations of this study

This study focused on systematic reviews of diagnostic test accuracy studies. Therefore, we do not know what the implications are for other types of systematic reviews. However, the methods in this study are applicable to systematic reviews estimating numerical values, and our results may therefore be applicable also to systematic reviews of interventions.

Due to the nature of the datasets, we could only recalculate meta-analyses using data from studies indexed in PubMed. Previous studies examining the impact of only searching PubMed on meta-analyses of interventions demonstrated moderate changes in estimates, and observed changes were equally likely to favor controls as interventions [22, 24]. In this study, we assume that searching only PubMed is similarly unbiased for diagnostic test accuracy, but we are aware of no studies examining this directly. Limiting the meta-analyses to PubMed does however reduce the number of studies available for analysis and may therefore mean that we are underestimating the applicability of these stopping criteria and that we may be observing greater variance than we would in a prospective setting.

This study focused on Cochrane systematic reviews, which are known to have higher consistency and lower bias than other systematic reviews [52]. It is not clear what the implications are for systematic reviews conducted with less stringency than Cochrane systematic reviews.

The definition of loss we use for evaluation (*L*_2_) makes the simplifying assumption that sensitivity and specificity are equally important. Specificity values of diagnostic tests tend towards values close to one and thus often exhibit smaller variance than the sensitivity. As a result, the *L*_2_ loss is often dominated by the sensitivity loss (Table 1). We also report loss separately for sensitivity and specificity in our analysis.

### Future work

Future work will evaluate the validity of these results in prospective settings. We also plan to use Bayesian methods to estimate final meta-analysis accuracy from the study data accumulated through the screening process. Furthermore, we will also aim to extend this approach to other study types beyond diagnostic test accuracy, such as intervention studies.

## Conclusions

Our results suggest that diagnostic summary sensitivity and specificity can be estimated within an accuracy of 2 percentage points while deliberately missing over 40% of the relevant studies within a single database. This is contrary to current guidelines which assume that an exhaustive search is necessary to produce reliable estimates with low bias. On the other hand, we find a clear relationship between the absolute size of the meta-analysis and the reliability and precision of the estimates. In other words, a reliable meta-analysis requires identifying a sufficient number of studies, but how large a fraction of relevant studies is identified is less important.

In the simulations, a combination of stopping criteria reduced the screening effort by 71.2% on average (median 86.8%, range 0% to 99.93%) for the main meta-analysis in each systematic review and triggered in every systematic review with more than 1000 candidate studies. No systematic review required screening more than 2,308 studies, whereas exhaustive manual screening required screening up to 43,363 studies. Despite an average 70% recall, the estimation error was 1.3% on average, much less than the estimation error expected when replicating summary estimate calculations.

The (retrospective) 95% recall criterion yielded an average 0.1% error when ranking with logistic regression and an average 0.2% error when using random order. Thus, we confirm the hypothesis that 95% recall is sufficient to accurately estimate the main meta-analysis in systematic reviews of diagnostic test accuracy, provided the ranking method is unbiased. On the other hand, we observe almost unchanged estimates (within 2% tolerance) for recall as low as 30%, and 95% recall is thus not necessary to reach accurate estimates.

## Data Availability

Thefirst dataset supporting the conclusions of this article is available in the Zenodo repository (DOI: 10.5281/zenodo.1303259, https://zenodo.org/record/1303259#.XBpPGMZ7kUE), the second is available from github (https://github.com/CLEF-TAR/tar).

## Abbreviations

clef: Conference and Labs of the Evaluation Forum, formerly known as the Cross-Language Evaluation Forum
dta: Diagnostic test accuracy
loocv: Leave-one-out cross-validation
ma: Moving average. We further denote moving average with different window sizes by MA2, MA3, MA4, etc.
